# Infective endocarditis presenting with myocardial infarction with non-obstructive coronary arteries: A case report and literature review

**DOI:** 10.1097/MD.0000000000050707

**Published:** 2026-09-18

**Authors:** Lan Cheng, Lianjie Li

**Affiliations:** a Department of Cardiovascular Medicine, Ningbo No. 2 Hospital, Wenzhou Medical University, Ningbo, Zhejiang, P.R. China; b Department of Neurosurgery, Ningbo No. 2 Hospital, Wenzhou Medical University, Ningbo, Zhejiang, P.R. China.

**Keywords:** acute coronary syndrome, case report, coronary angiography, infective endocarditis, literature review, myocardial with nonobstructive coronary arteries

## Abstract

**Rationale:**

Infective endocarditis (i.e.) results from the combined effects of multiple factors, such as frequent vascular access procedures and impaired immune system function, and is relatively common among patients with uremia undergoing long-term hemodialysis. Myocardial infarction with nonobstructive coronary arteries (MINOCA) may present as an atypical clinical presentation, especially in patients with multiple comorbidities.

**Patient Concerns:**

We report a rare case of a 52-year-old male who underwent regular hemodialysis using a tunneled-cuffed catheter and presented with persistent acute chest distress and pain for 4 hours.

**Diagnoses:**

Immediately after defecation, the patient developed persistent severe chest pain, shortness of breath, and profound sweating. ST-segment depression and T-wave alterations in the inferior leads (II, III, and aVF) on the initial ECG, together with elevated troponin I levels, raised suspicion of acute myocardial infarction (AMI). Emergency coronary angiography showed an irregular aortic valve, no significant stenosis of the left main coronary artery, 20–30% stenosis of the middle segment of the anterior descending artery, 30–40% stenosis of the circumflex artery, and no significant stenosis of the right coronary artery. Laboratory results revealed an increased percentage of white blood cells, neutrophils, and C-reactive proteins (CRP). Cardiac ultrasonography revealed hyperechogenicity of the aortic valve, suggesting the presence of vegetation. Considering the patient’s history of uremia, long-term hemodialysis, and the absence of obstructive coronary arteries, MINOCA secondary to IE was suspected.

**Interventions:**

Treatment with broad-spectrum antibiotics was initiated. Given the echocardiographic findings of massive and moderate aortic valve regurgitation, the patient underwent aortic valve replacement with cardiopulmonary bypass.

**Outcomes:**

The patient’s inflammatory markers and white blood cells significantly decreased, serum troponin decreased, and ST-T changes in the ECG significantly recovered. After mechanical aortic valve replacement, the patient was discharged with anti-infection therapy, cardiac strengthening, blood purification, and nutritional support.

**Lessons:**

The clinical presentation of infective endocarditis is atypical in patients undergoing dialysis, especially in the presence of other life-threatening conditions. Early multimodal imaging and multidisciplinary collaboration are essential for the timely diagnosis and management of MINOCA and IE.

## 1. Introduction

Infective endocarditis (IE) affects the inner lining of the heart, particularly involving the cardiac valves and the endocardium, and is associated with high morbidity and mortality.^[1]^ Epidemiological studies have shown that the incidence of IE has increased worldwide. A recent observational study estimated 264,282 incident cases of IE in China in 2021, with an age-standardized incidence rate of 13.48%.^[2]^ Left-sided IE is more common than right-sided native valve IE, with the aortic and mitral valves being the most frequently affected.^[3]^ Approximately 20–40% of patients with IE experience embolic events, most commonly involving cerebral embolism.^[4]^ In contrast, coronary artery embolism is rare but has a high mortality rate.^[5]^ This phenomenon may result in acute myocardial infarction (AMI), a rare but potentially fatal complication of IE.^[6]^ Studies have indicated that dialysis patients using central venous catheters have a more than two-fold higher risk of developing IE than those using arteriovenous fistulas.^[7]^ Myocardial infarction with nonobstructive coronary arteries (MINOCA) is considered a diagnostic approach that is clinically adaptable and needs further investigation, rather than a fixed diagnosis. MINOCA is associated with an increased risk of mortality, recurrent angina, and adverse socioeconomic costs. Proposed mechanisms include coronary artery spasm, spontaneous coronary artery dissection, plaque disruption or erosion, coronary embolism, and microvascular dysfunction.^[8]^

Herein, we report a case of MINOCA combined with IE in a chronic hemodialysis patient. Our aim is to raise awareness among young clinicians of the importance of conducting prompt imaging examinations and appropriate treatment.

## 2. Case presentation

This study was approved by the Ethics Committee of Ningbo No. 2 Hospital. Written informed consent was obtained from the patient and his family members for publication of this case report and the accompanying images. The requirement for additional IRB review was waived in accordance with institutional policy. We report the case of a 52-year-old male who presented with sudden-onset chest tightness and severe pain for 4 hours after defecation. The pain was severe, persistent, and intolerable, accompanied by shortness of breath and profuse sweating. The patient denied dizziness, blurred vision, nausea, vomiting, or numbness of the upper limbs. He was brought to the emergency department (ED) by his family.

The patient had a 10-year medical history of chronic renal failure requiring hemodialysis, as well as uncontrolled diabetes mellitus and primary hypertension. He had no history of smoking, alcohol consumption, or use of other psychoactive substances, and no history of trauma or pseudogout. On presentation, his vital signs were within normal limits except for a low-grade fever (37.6°C) and a higher pulse rate (103 beats/min). His BMI was 15.62 kg/m^2^.

On physical examination, the superficial lymph nodes were not swollen on palpation and there were no specific rashes. The heart, lungs, and abdomen showed no abnormalities. Cardiac auscultation revealed a diastolic aortic murmur at the left sternum border. No abnormalities were observed in the lungs or the abdomen. No significant edema was observed in the lower limbs.

**Figure 1.**
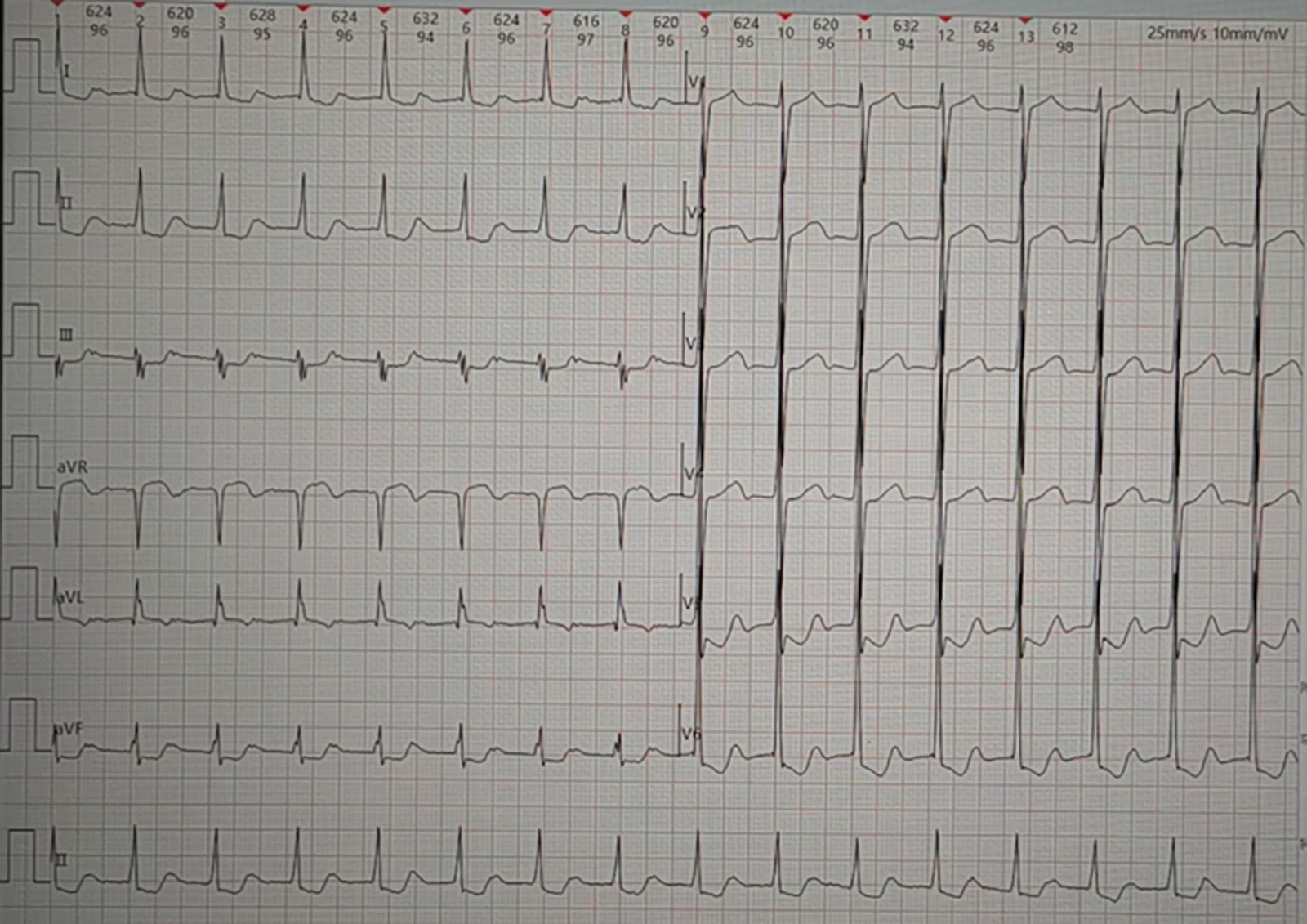
Electrocardiogram at admission showed ST depression in the inferior leads.

**Figure 2.**
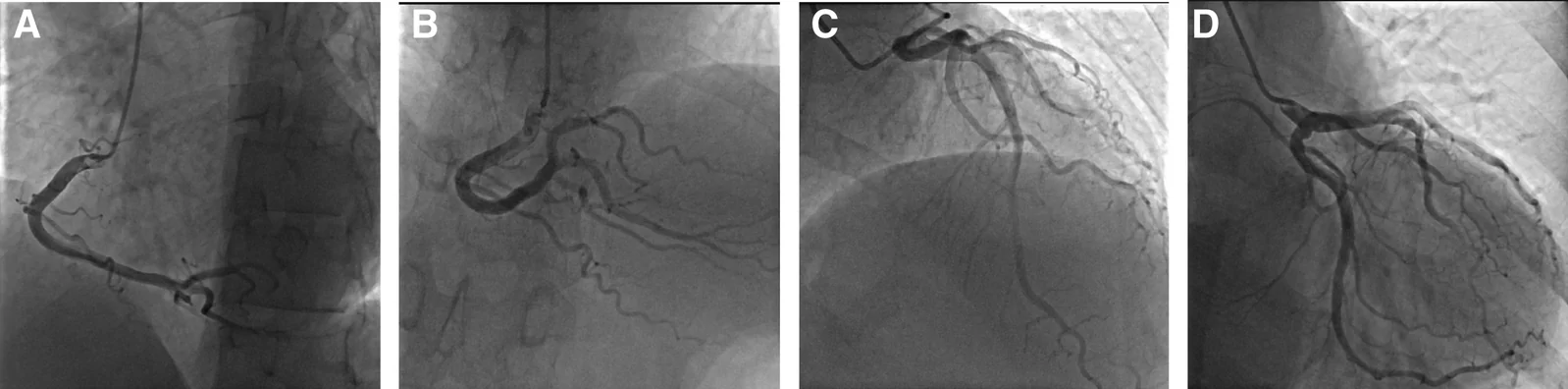
Coronary angiography shows blood flow in major large vessels. (A, B) Angiography of the proximal, middle, and distal segments of the right coronary artery; (C, D) Angiography of the proximal, middle, and distal segments of the left coronary artery.

**Figure 3.**
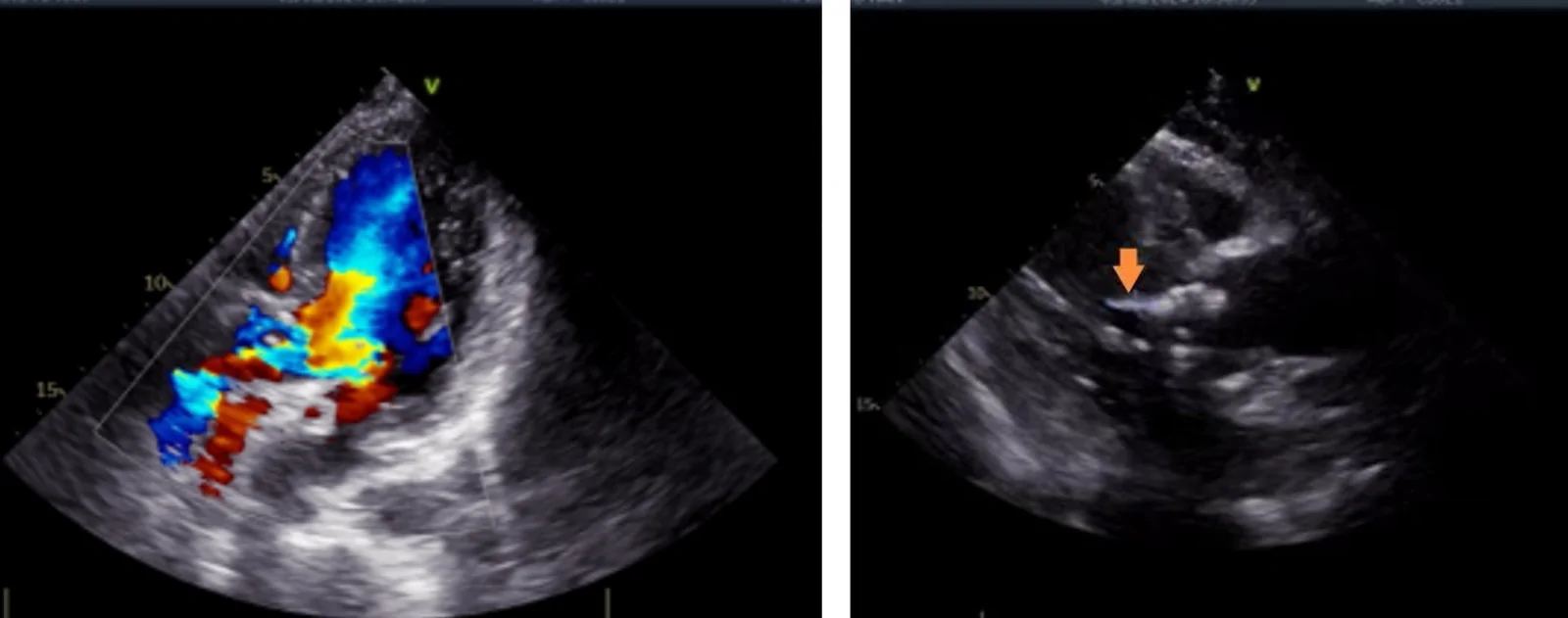
Echocardiography revealed massive aortic regurgitation with moderate stenosis and suspected vegetations. Yellow arrows represent suspicious vegetations.

Upon admission ECG showed ST-segment depression and T-wave alteration in the inferior leads (II, III, and aVF), combined with an increased cTnI (>356 ng/L), indicating NSTEMI (Fig. 1). Because the patient had persistent severe chest pain and a Global Registry of Acute Coronary Events (GRACE) risk score of 189 (high risk), he received a loading dose of aspirin (300 mg) and clopidogrel (600 mg), together with rosuvastatin (20 mg), and underwent emergency coronary angiography. Coronary angiography revealed an irregular aortic valve, no significant stenosis in the left main coronary artery, 20–30% stenosis in the middle segment of the anterior descending artery, 30–40% stenosis in the circumflex artery, and no significant stenosis in the right coronary artery (Figs. 2A–D). Transthoracic echocardiography (TTE) revealed aortic valve vegetation combined with severe and moderate aortic regurgitation (Fig. 3). Laboratory results were as follows: white blood cell count, 23900/μL; hemoglobin, 113 g/L; platelets, 40.2 × 10^4^/μL; hypersensitive CRP, 112.01 mg/L (reference range: ≤6 mg/L); and erythrocyte sedimentation rate (ESR), 48 mm/h (reference range: ≤20 mm/h). Emergency renal function tests showed the following results: creatinine, 821.1 μmol/L; urea, 16.39 mmol/L; and B-type natriuretic peptide (BNP), 3069 pg/ml. The remaining parameters, including thyroid and coagulation functions, were within normal ranges.

Embolization of the small coronary vessels due to septic embolism was first considered. After collecting blood culture samples several times, empirical antibiotics (piperacillin, sodium, and linezolid) were initiated upon admission. Blood cultures finally revealed the presence of methicillin-resistant staphylococcus aureus (MRSA).

On the 14th day of admission, the patient’s body temperature was within the normal range, inflammatory markers were obviously reduced, and the patient underwent aortic valve replacement with the assistance of cardiopulmonary bypass. Anti-infection and multi-organ support contributed to his recovery. In addition, there was no recurrent chest discomfort, he achieved a 6-minute walk test of 480 m, and he had no signs of heart failure after 1 month of follow-up. Troponin level returned to normal, and physical examination confirmed that the typical high-pitched diastolic murmur of aortic regurgitation, which was detected preoperatively, disappeared completely. Secondary prevention medication was well tolerated after 2 months of follow-up. The details of this case are arranged in a timeline shown in Table 1.

**Table 1:** Timeline in this case.

Patient past medical history	End-stage renal disease on regular hemodialysis via indwelling venous catheters.
July 31, 2024 (Admission)	a 52-year-old male, who complained of sudden chest tightness and pain 4 hours after defecation; ECG upon admission showed ST-segment depression and T-wave alteration in the inferior leads (II, III, and aVF) combined with increased cTnI (>356ng/L). Physical examination detected a diastolic aortic murmur on the left border of the sternum.
August 1, 2024	Coronary angiography performed, confirmed no obstructive coronary lesions, Laboratory results were as follows: white blood cell count, 23900/μL; hemoglobin 113 g/L; platelets, 40.2 × 10^4^/μL; hypersensitive CRP, 112.01 mg/L (normal range: ≤6); and ESR,48 mm/h (normal range: ≤20). Emergency renal function showed the following results: creatinine, 821.1μmol/L; urea, 16.39 mmol/L; and BNP, 3069 pg/ml, regurgitation diagnosed as MINOCA with i.e.. Initiated dual antiplatelet, anti-ischemia therapy and antibiotic treatment. The patient was admitted to CCU.
August 5, 2024	Chest pain and dyspnea completely resolved, vital signs remained stable for 48 hours. The patient was transferred from CCU to the general cardiology ward for subsequent routine rehabilitation and secondary prevention management, TTE revealed aortic valve vegetation combined with severe and moderate aortic valve regurgitation with decreased left ventricular ejection fraction. After 2 sets of peripheral blood cultures were collected before antibiotic initiation, the empirical antibiotic regimen was adjusted according to the local antimicrobial resistance profile and the patient’s clinical presentation.
September 10, 2024	Body temperature returned to normal range, and inflammatory markers showed a marked decline compared with prior levels. After completing presurgical cardiac function assessment and anticoagulation bridging preparation, the patient was transferred to the Department of Cardiac Surgery for elective aortic mechanical valve replacement surgery.
October 15, 2024 Follow-up	No recurrent chest discomfort, 6-minute walk test reached 480m, no signs of heart failure.
November 15, 2024 Follow-up	Troponin level returned to normal, physical examination confirmed that the typical high-pitched diastolic murmur of aortic regurgitation, which was detected preoperatively, disappeared completely, secondary prevention medication well tolerated.

## 3. Discussion

Severe IE is often accompanied by heart failure and systemic embolic events, often requiring multidisciplinary team management and resulting in high mortality. Approximately 2–6% of patients undergoing long-term hemodialysis develop IE, representing a risk that is estimated to be 50–60 times higher than that in the general population. Several factors contribute to this increased susceptibility. First, frequent vascular access procedures, particularly the use of central venous catheters in hemodialysis patients, increase the risk of bacterial invasion, leading to a significantly higher incidence of IE.^[9]^ Studies have indicated that dialysis patients using central venous catheters have a more than two-fold higher risk of developing IE than those using arteriovenous fistulas.^[7]^

Furthermore, the impaired immune system function in dialysis patients renders them more susceptible to infections.^[10]^ Among these patients, the pathogens causing IE are predominantly gram-positive bacteria, particularly *Staphylococcus aureus*, which is associated with higher infection and mortality rates.^[11]^ Studies have indicated that the in-hospital mortality rate of IE in dialysis patients reaches 50%, and this proportion is even higher among patients with vascular access infections.

Septic embolization frequently complicates IE, with systemic embolization often occurring in left-sided IE, which can lead to stroke, vision loss, or splenic infarction.^[12]^ Coronary artery septic embolism is uncommon, with a reported incidence of 0.31–0.51% among patients with IE, but it is associated with low morbidity and poor survival rates.^[13]^ A previous case report described a 62-year-old woman undergoing regular hemodialysis via an indwelling venous catheter who was finally diagnosed with pulmonary embolism and inferolateral ST-elevation myocardial infarction.^[14]^ Myocardial infarction secondary to septic coronary embolism is a rare but potentially life-threatening complication of IE. Prompt diagnosis and antibiotic therapy could decrease the risk of multiple systemic embolization. Therefore, a high degree of suspicion should be entertained when examining patients presenting with specific cardiac symptoms, increased troponin levels, ECG alterations, and a previous medical history of endocarditis.

Moreover, guidelines propose the use of dual antiplatelet therapy in conjunction with anticoagulation for AMI, which was consistent in this case. However, the European Society for Cardiology has issued a recent update to its ACS management guidelines, in which treatment with P2Y12 antagonists had been downregulated.^[15]^ It is certainly important in cases where patients initially present with ACS resulting from IE and combined with chronic kidney disease (CKD). Patients with CKD have a substantially higher long-term bleeding risk than the general population. Although antiplatelet therapy is often indicated in patients with suspected acute coronary syndrome, it may further increase the risk of bleeding in this population. Therefore, when i.e. is strongly suspected, emergency TTE should be conducted to facilitate timely diagnosis and guide appropriate management.

MINOCA, which belongs to a special subtype in the classification of myocardial infarction, has recently gained increasing recognition. It is characterized by evidence of acute myocardial infarction when coronary angiography shows that the degree of stenosis of the major epicardial vessels is <50% and there is no clear obstructive coronary artery disease. Approximately 7–15% of patients presenting with ASC have no evidence of obstructive coronary artery disease on coronary angiography.^[16–18]^ Current ESE guidelines recommend cardiac magnetic resonance imaging (MRI) as a key diagnostic modality for identifying the underlying cause of MINOCA.^[19]^ Clinicians diagnose MINOCA mainly based on the results of coronary angiography and clinical symptoms and laboratory tests. In the PROMISE clinical trial, multiple techniques have been proposed to help diagnose MINOCA, such as optical coherence tomography (OCT), intracoronary acetylcholine provocation, and microRNA sampling.^[19]^

In the present case, the patient’s clinical features and laboratory findings were highly consistent with NSTEMI. However, because emergency coronary angiography revealed no significant obstructive coronary artery disease, he was diagnosed with MINOCA accompanied by IE. One possible explanation is that although emergency coronary angiography did not reveal embolization of a major coronary artery, obstruction of a small terminal branch cannot be ruled out. This is because coronary angiography primarily evaluates the normality, stenosis, and plaque of the main trunk and the large branches of the large and middle vessels. Second, systemic inflammation, toxin release, and sympathetic nerve stimulation caused by IE induce diffuse coronary artery spasms, temporary constriction of the lumen, and interruption of blood flow. In the current case, after the coronary artery was relieved, angiography was repeated, and the blood vessels returned to normal without any changes. A third possible mechanism is that the aortic valve vegetation covers the opening of the coronary artery, leading to AMI. In our patient, the angiography catheter was slightly displaced or angulated and did not get stuck in the narrow opening. Furthermore, the main coronary artery appeared to be patent. Microangiopathy, myocardial microcirculation infarction, IE caused by inflammation, sepsis, and endothelial injury may contribute to myocardial microvascular dysfunction; whilst large coronary blood flow is not affected, the microcirculation blood supply is interrupted, which can lead to the clinical manifestation of AMI. It is therefore important to identify other diseases with symptoms of myocardial infarction in clinical practice; key points are shown in Table 2.

**Table 2:** Differential diagnosis: myocardial infarction with normal coronary angiogram.

Disease	Key Distinguishing Features
Infective Endocarditis (i.e.)	Fever, heart murmur, valvular vegetation, positive blood culture, multiple systemic embolisms
Coronary Artery Spasm	No fever, no valvular murmur, no infectious or toxic symptoms
Stress Cardiomyopathy (Takotsubo Cardiomyopathy)	Triggered by emotional/physical stress, typical apical ballooning morphology, no fever or infection
Coronary Microvascular Disease	No fever, no valvular vegetation, chronic chest tightness and shortness of breath
Aortic Dissection Involving Coronary Ostia	Severe tearing chest pain, asymmetric blood pressure, confirmed by CTA

The treatment of IE in patients with MINOCA continues to be a challenging clinical issue for clinicians. Although antibiotic therapy is the first-line approach, cardiac surgery may be necessary for patients with severe comorbidities. Previous studies have demonstrated that cardiac surgery significantly reduces long-term mortality in IE patients.^[20]^ Furthermore, emergency TTE may play a pivotal role in facilitating early diagnosis and avoiding unnecessary examinations and treatment for patients to obtain the best treatment time, reduce adverse reactions, and improve prognosis.

This case report has several limitations. First, cardiac MRI was not performed because of technical limitations; therefore, direct imaging evidence of myocardial infarction was unavailable. Second, as this type of case history is rare, the findings cannot be readily generalized to a broader patient population. We speculated that septic embolism derived from the aortic valve contributed to myocardial microcirculation infarction; however, direct evidence supporting this mechanism was unavailable.

In conclusion, this case revealed the rarity of MINOCA accompanied by IE in a patient with end-stage kidney disease undergoing routine hemodialysis via an indwelling venous catheter. This case places considerable emphasis on the initial diagnosis and prompt treatment in patients presenting with ACS combined with IE to decrease the risk of septic embolism dissemination and ensure better outcomes.

## Acknowledgments

This study was funded by the Project of NINGBO Leading Medical & Health Discipline (Project Number: 2026-A29) and the Medical and Health Science Program of Zhejiang Province (Grant No.2025KY1388). We would also like to thank Editage (www.editage.cn) for English language editing.

## Author contributions

**Data curation**: Lan Cheng.

**Visualization**: Lianjie Li.

**Writing – original draft:** Lan Cheng.

**Writing – review & editing:** Lianjie Li.
